# OTEH-6. Algorithmic approach to characterize post-treatment recurrent glioma using RNA sequencing and quantitative histopathology

**DOI:** 10.1093/noajnl/vdab070.045

**Published:** 2021-07-05

**Authors:** Michael Argenziano, Akshay Save, Deborah Boyett, Jack Grinband, Hyunsoo Yoon, Jing Li, Matei Banu, Guy McKhann, Michael Sisti, Kristin Swanson, Jeffrey Bruce, Peter Canoll

**Affiliations:** 1 Columbia University Vagelos College of Physicians and Surgeons, New York, NY, USA; 2 Department of Neurological Surgery, CUMC, New York, NY, USA; 3 NYU Langone Health, New York, NY, USA; 4 Department of Neurology, CUMC, New York, NY, USA; 5 Thomas J. Watson College of Engineering and Applied Science, SUNY Binghamton, Binghamton, NY, USA; 6 Milton Stewart School of Industrial and Systems Engineering Georgia Institute of Technology, Atlanta, GA, USA; 7 Mathematical NeuroOncology Lab, Mayo Clinic Arizona, Phoenix, AZ, USA; 8 Department of Pathology and Cell Biology, New York, NY, USA

## Abstract

**Introduction:**

Distinguishing between tumor and treatment effect in post-treatment glioma, although crucial for clinical management, is difficult because contrast-enhancing regions are mixtures of recurrent tumor and reactive tissue, and definitive histopathological criteria do not exist. This study disentangles the marked intra-tumoral heterogeneity in the treatment-recurrent setting by developing an unsupervised framework to algorithmically categorize intraoperative MRI-localized biopsies into three clinically-relevant tissue clusters based on joint analysis of RNA sequencing and histopathological data.

**Methods:**

A retrospective cohort of 84 MRI-localized biopsies from 37 patients with post-treatment recurrent glioblastoma underwent mRNA extraction and quantification via PLATEseq protocol. For 48 of 84 biopsies, a neighboring piece of tissue underwent quantitative histopathology based on labeling index (LI) for SOX2, CD68, NeuN, Ki67, and H&E. Correlation between LIs and gene expression for these 48 samples was performed. Genes significantly correlated (p<0.05) with ≥1 marker were used for hierarchical clustering of correlation matrix, identifying three mutually-exclusive tissue-specific gene sets. These sets were then used to perform ssGSEA to categorize each of 84 biopsies into one of three tissue types.

**Results:**

Correlation analysis identified 7779 genes significantly correlated with ≥1 histopathological marker. Clustering revealed three gene sets associated with specific markers: SetA-3688 genes associated with SOX2/Ki67/H&E; SetB-2418 genes associated with CD68; SetC-1673 genes associated with NeuN. ssGSEA using these sets categorized each biopsy into one of three tissue types: 27 biopsies enriched in SetA, 28 in SetB, and 29 in SetC.

**Conclusions:**

Using MRI-localized biopsies with both RNAseq and histopathological data, this algorithmic approach allows development of three orthogonal tissue-specific gene sets that can be applied to characterize the heterogeneity in post-treatment recurrent glioma: SetA: genes correlated with SOX2/Ki67/H&E, representing recurrent tumor; SetB: genes correlated with CD68 (microglia) representing reactive tissue consistent with treatment effect; SetC: genes correlated with NeuN (neurons), representing infiltrated brain.

